# Identification of efferocytosis-related subtypes in gliomas and elucidating their characteristics and clinical significance

**DOI:** 10.3389/fcell.2023.1295891

**Published:** 2023-12-13

**Authors:** Mengge Gao, Jinsheng Huang, Bo Yang, Qiong Liu, Miaoqing Luo, Biying Yang, Xujia Li, Xiaofang Liu

**Affiliations:** ^1^ Department of Clinical Nutrition, Huadu District People’s Hospital of Guangzhou, Southern Medical University, Guangzhou, China; ^2^ State Key Laboratory of Oncology in South China, Sun Yat-sen University Cancer Center, Guangzhou, China

**Keywords:** glioma, effercytosis, personalized treatment, subtype, immune landscape, metabolism, intercellular communication

## Abstract

**Introduction:** Gliomas, the most prevalent tumors of the central nervous system, are known for their aggressive nature and poor prognosis. The heterogeneity among gliomas leads to varying responses to the same treatments, even among similar glioma types. In our study, we efferocytosis-related subtypes and explored their characteristics in terms of immune landscape, intercellular communication, and metabolic processes, ultimately elucidating their potential clinical implications.

**Methods and Results:** We first identified efferocytosis-related subtypes in Bulk RNA-seq using the NMF algorithm. We then preliminarily demonstrated the correlation of these subtypes with efferocytosis by examining enrichment scores of cell death pathways, macrophage infiltration, and the expression of immune ligands. Our analysis of single-cell RNA-seq data further supported the association of these subtypes with efferocytosis. Through enrichment analysis, we found that efferocytosis-related subtypes differ from other types of gliomas in terms of immune landscape, intercellular communication, and substance metabolism. Moreover, we found that the efferocytosis-related classification is a prognostic factor with robust predictive performance by calculating the AUC values. We also found that efferocytosis-related subtypes, when compared with other gliomas in drug sensitivity, survival, and TIDE scores, show a clear link to the effectiveness of chemotherapy, radiotherapy, and immunotherapy in glioma patients.

**Discussion:** We identified efferocytosis-related subtypes in gliomas by analyzing the expression of 137 efferocytosis-associated genes, exploring their characteristics in immune landscape, intercellular communication, metabolic processes, and genomic variations. Moreover, we discovered that the classification of efferocytosis-related subtypes has a strong prognostic predictive power and holds potential significance in guiding clinical treatment.

## Introduction

Gliomas, the most predominant subtype of brain neoplasms, constitute 23.3% of all central nervous system (CNS) tumors and account for a substantial 78.3% of malignant CNS tumors (Molinaro et al., 2019; Sung et al., 2021). Among all primary malignant tumors, glioblastoma (GBM) exhibits the highest incidence, accounting for approximately 48.6% of all gliomas (Sung et al., 2021). Given the high-degree malignancy and aggressive invasiveness of glioblastoma, the prognosis for patients with glioblastoma is poor. Even when treated with the STUPP protocols and tumor-treating fields (TTF) therapy, the median survival time (mOS) for patients remains a mere 15–18 months (Stupp et al., 2005; Kotecha et al., 2023). The prognosis for patients with LGG (Low-Grade Glioma) is generally more favorable. The mOS for patients with grade II and grade III gliomas is 11 years and 3 years, respectively (Smoll et al., 2012). Currently, therapeutic approaches for gliomas consist mainly of surgical resection, combined with chemoradiotherapy, targeted therapy and TTF. The discovery of immune-related targets and the observed effectiveness of immunotherapy hold promising potential for the treatment of gliomas (Yang et al., 2022). However, identical therapeutic approaches result in variable treatment responses even among similarly categorized gliomas. A retrospective study showed that bevacizumab can improve the prognosis of patients with proneural subtype GBM or IDH wild-type GBM, compared with those with other subtypes (Sandmann et al., 2015). Additionally, clinical research indicates that the therapeutic efficacy of pembrolizumab, an anti-programmed cell death protein 1 (PD1) antibody, is confined to glioma patients with specific types of DNA mismatch repair deficiencies (Yang et al., 2022). Therefore, with the continuous advancement in oncological therapies, identifying appropriate biomarkers to guide personalized treatment for glioma patients remains a critical endeavor. Signatures based on transcriptomic data have been developed and are used clinically, including the PAM50 classification for breast cancer, and the Phillips and Verhaak classifications for glioma (Phillips et al., 2006; Parker et al., 2009; Verhaak et al., 2010). Thus, utilizing transcriptomic data to identify specific biomarkers or subtypes is a feasible approach to guide treatment strategies.

During the progression of cancer, tumor cells can undergo various forms of cell death, such as apoptosis, necroptosis, ferroptosis, and pyroptosis, due to mutations, hypoxia, and treatments like radiotherapy and chemotherapy (Chen et al., 2021). During apoptosis, cells release the “find me” signal, such as C-X3-C motif chemokine ligand 1 (CX3CL1), to recruit macrophages. These macrophages then adhere to ‘eat me’ signals on the apoptotic cells, including phosphatidylserine and calreticulin, either directly or indirectly through mediators like Protein S and growth arrest-specific protein (GAS6) (Boada-Romero et al., 2020). This process is referred to as efferocytosis. Meanwhile, normal cells suppress phagocytosis by macrophages through the expression of ‘don’t eat me’ signals, such as CD47 and MHC-I.

The process of efferocytosis can help to create an immune-suppresive microenvironment, thus assisting tumor immune escape (Thorsson et al., 2018). The efferocytosis of apoptotic cells drives macrophage M2 polarization and generates anti-inflammatory mediators, thereby contributing to this immuno-suppressive environment. Additionally, the products of non-infected apoptotic cells are transported to recycling endosomes instead of MHC class II-loading compartment, thus preventing the presentation of antigens derived from apoptotic cells. In glioblastoma, blocking the MerTk receptor, which binds to apoptotic cells via protein S/GAS6, can induce a pro-inflammatory immune microenvironment and reduce the infiltration of M2 macrophages within the tumor (Wu et al., 2018). Additionally, combining anti-T-cell immunoglobulin mucin 3 (TIM3) antibody, radiotherapy, and anti-PD-1 therapy has been shown to enhance antitumor responses in mice (Kim et al., 2017). Therefore, identifying gliomas with a high level of efferocytosis may aid in selecting patients who could benefit from efferocytosis-targeted immunotherapies, potentially improving their prognosis.

**FIGURE 1:**
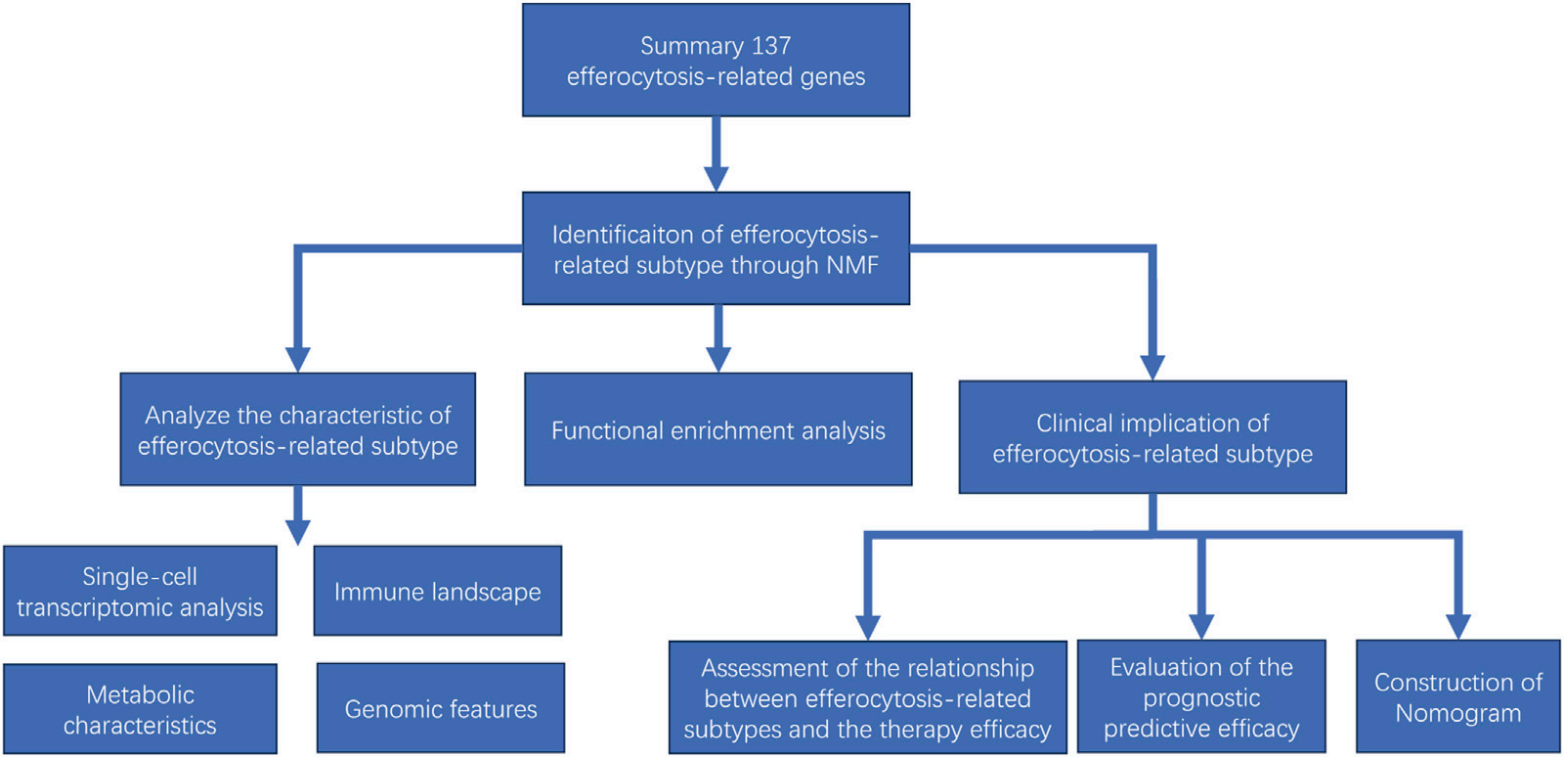
Flow chart showing the study procedure.

The aim of this study is to identify efferocytosis-related subtypes in gliomas through transcriptomic data analysis and elucidate their distinctive features in terms of immune landscape, intercellular communication, substance metabolism and genomic variation. Furthermore, our research also analyzes the correlation between the efferocytosis-related classification and patient prognosis, as well as the efficacy of radiotherapy and immunotherapy, aiming to provide novel insights for guiding personalized treatment and prognostic prediction. An overview of our study’s workflow can be found in Figure 1.

## Materials and methods

### Data collection and processing

The Cancer Genome Atlas (TCGA) and the Chinese Glioma Genome Atlas (CGGA) provided comprehensive datasets that include copy number variation (CNV), transcriptome sequencing, somatic mutations and clinical information (Zhao et al., 2021). We retrieved a dataset from Synapse, comprising bulk and single-cell transcriptomic data from eight paired glioma patient samples (accessible at Synapse:syn22257780) (Johnson et al., 2021). The immune-related data used in this study, including immune molecules and TCR Shannon, were obtained from the Supplementary Material of the publication ‘The Immune Landscape of *Cancer* (available at https://doi.org/10.1016/j.immuni.2018.03.023) (Thorsson et al., 2018). The Gene-Expression Omnibus (GEO) database also provided microarray gene expression data and clinical records for three glioma datasets (Rembrandt, Gravendeel, and Kamoun cohorts). Patients with a survival duration of less than 30 days were intentionally excluded from the analysis. To evaluate relative gene expression in gliomas, we converted raw count transcriptome data into transcripts per million (TPM) values.

### Identification of the efferocytosis-related subtype in glioma

137 efferocytosis-related genes (ERGs) were extracted from previous reviews (Supplementary Table S1) (Boada-Romero et al., 2020; Doran and Yurdagul, 2020). Subtypes were identified using unsupervised non-negative matrix factorization (NMF) algorithm in TCGA dataset (Gaujoux and Seoighe, 2010). The optimal number of clusters was determined by the first value that the cophenetic coefficients start decreasing according to the ‘NMF’ R package. Survival differences among patients with different glioma subtypes were analyzed using Cox regression analysis and visualized through Kaplan-Meier (KM) survival curves. The Timer2.0 web server (accessible at http://timer.cistrome.org/) was used to evaluate the degree of macrophage infiltration differences between different glioma subtypes (Li et al., 2020).

### Selection of method for classifying samples in external datasets

We divided the TCGA dataset into training and test sets at a 70:30 ratio using the ‘caret’ package. The training set was then trained using a suite of methods including PAM, Random Forest, SVM, Boruta, XGBoost, Lasso regression, and univariate Cox analysis. Subsequently, we predicted the test set to compute the area under the curve (AUC) values.

### Processing and analysis of single-cell RNA sequencing data

The SCTransform function in the ‘Seurat’ package was used to normalize counts for each glioma sample. Then the ‘Harmony’ package was applied to integrate the different samples following principal component analysis (PCA). For dimensional reduction and visualization, we utilized Uniform Manifold Approximation and Projection (UMAP). The primary cell types of the single-cell transcriptomic dataset were annotated following the original authors’ classification (Johnson et al., 2021). The ‘CellChat’ package was used to analyze intercellular communication in C1 and C2 (Jin et al., 2021). We applied Gene Set Enrichment Analysis (GSEA) to evaluate pathway enrichment differences between the C1 and C2 subtypes within the same cell type.

### Functional enrichment analysis

To identify differentially expressed genes (DEGs) between C1 and C2, the R package ‘Deseq2′ was used (log2FC > 1 and *p* <0.05). The ‘ClusterProfiler’ package was used to annotate the function of DEGs using Gene Ontology (GO) and Kyoto Encyclopedia of Genes and Genomes (KEGG) analyses (Yu et al., 2012). We also applied GSEA to explore regulatory differences in GO gene sets and KEGG pathways between C1 and C2. The ClueGO plugin in Cytoscape was utilized to simplify and visualize the pathways enriched in the C1 subtype as determined by GSEA (Bindea et al., 2009).

### Evaluation of the immune landscape

The ESTIMATE score, stromal score, and immune score of each glioma sample were calculated using the ‘Estimate’ package (Yoshihara et al., 2013). We obtained a list of 75 immunomodulatory genes previously summarized in the literature (Thorsson et al., 2018). The results from the Tumor Immune Estimation Resource (TIMER) and CIBERSORT-ABS were used to assess the infiltration of various types of immune cells in gliomas. The Exclusion and Dysfunction scores of TCGA dataset were downloaded from the Tumor Immune Dysfunction and Exclusion (TIDE) website (accessible at http://tide.dfci.harvard.edu) (Jiang et al., 2018).

### Assessment of intercellular communication

Drawing on established methods for constructing immune networks, we developed immune regulation networks for C1 and C2 subtypes as follows. We began by selecting 41 ligands and receptors from a list of 75 immunomodulatory genes. Human protein-protein interaction data were then retrieved via CellChat to identify all genes that could interact with these 41 ligands and receptors as candidates. Next, we classified the expression levels of these genes within the TCGA dataset into high, medium, or low categories. Genes were entered into the immune network if at least 66% of samples showed mid or high expression levels. We then calculated a concordance index for each interacting pair as [(‘high’,'high')+(‘low’,'low')]/[(‘low’,'high')+(‘high’,'low')], with edges having a concordance index >1 being included in the network. Isolated nodes were ultimately removed to refine the network. Cytoscape was utilized to visualize the immune network. To identify hub immune molecules in the C1 immune network, we applied the Maximal Clique Centrality (MCC) and Degree algorithms. Intercellular communication at the single-cell level between C1 and C2 subtypes was analyzed using the ’'compareInteractions’' and ''netVisual_diffInteraction’' functions from the ‘CellChat’ package.

### Analysis of metabolism-related pathways

Seven pivotal metabolic pathways were curated from the literature and an additional four pathways related to energy metabolism were retrieved from the Molecular Signatures Database (MsigDB) to evaluate the energy metabolism of different subtypes (Peng et al., 2018; Yu et al., 2021). Gene Set Variation Analysis (GSVA) was then conducted to assess the enrichment of these 11 pathways across the subtypes (Hanzelmann et al., 2013).

### Genome analysis of different efferocytosis-related subtypes

Copy number segment (CNV) data downloaded from TCGA were utilized to identify CNV regions in gliomas via the GISTIC 2.0 pipeline. Mutation variation data for glioma was analyzed and visualized using the R package ‘maftools’. The predicted microsatellite instability (MSI) scores were calculated in the website http://tide.dfci.harvard.edu.

### Association between efferocytosis-related subtype and sensitivity to antineoplastic drugs

The imputed sensitivity score of 545 antineoplastic drugs from *Cancer* Therapeutics Response Portal (CTRP) for each glioma sample was calculated using the ‘OncoPredict’ package (Maeser et al., 2021). Additionally, we compared the imputed sensitivity score of eight chemotherapy regimens recommended in glioma treatment guidelines, which include six drugs, between the C1 and C2 clusters (Nabors et al., 2020). Correlations between the efferocytosis-related subtype and the imputed sensitivity score were analyzed via point-biserial correlation analysis.

### Association between different subtypes and immune checkpoint blockade (ICB) therapy

The TIDE score was calculated to estimate the response of patients to ICB therapy (accessible at http://tide.dfci.harvard.edu) (Jiang et al., 2018). Patients with a TIDE score greater than 0 are classified as non-responders to ICB therapy, whereas those with a score below 0 are considered likely to respond.

### Establishing and validating a nomogram

First, univariate Cox regression analysis was applied to determine the prognostic clinicopathological features of gliomas. Then, multivariate Cox regression was used to identify independent prognostic factors from these identified clinicopathological features. Independent prognostic factors were used to create a nomogram with the ‘rms’ R package. The predictive accuracy of the nomogram and other clinical features in the CGGA 325, and CGGA 693 datasets was assessed by the Area Under the Curve (AUC) using the ‘timeROC’ package.

### Statistical analysis

Data analysis and visualization were carried out using R software, version 4.0.4. The Wilcoxon rank-sum and Kruskal–Wallis rank sum tests were used to compare the continuous variables not fitting a normal distribution, including the TMB, CNV burden, MSI, immune score, and stromal score. We utilized the Chi-square test to compare the proportions of responders and non-responders to ICB therapy between the C1 and C2 clusters.

## Results

### Identification of efferocytosis-related subtype

**FIGURE 2:**
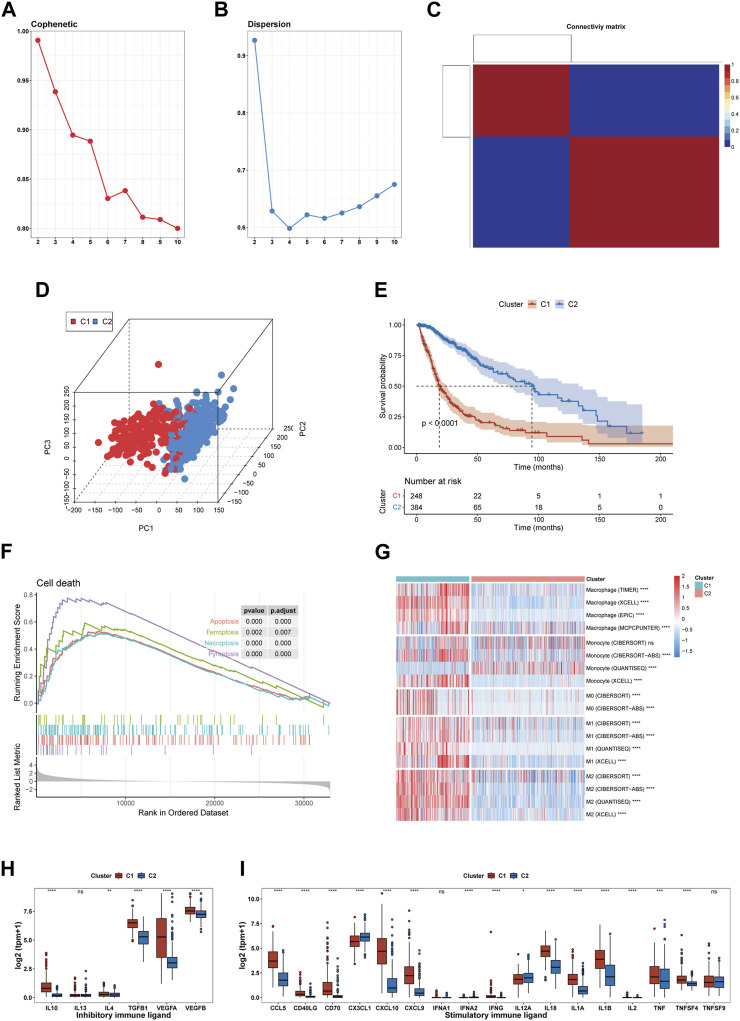
Identification of an efferocytosis-related glioma subtype in the TCGA cohort. **(A, B)** The cophenetic and dispersion coefficients of the NMF algorithm. **(C)** Connectivity matrix for glioma patients in the TCGA cohort by NFM when K = 2. **(D)** Three-dimensional PCA plot showing the distribution of C1 and C2. **(E)** Kaplan–Meier curve for patients in C1 and C2. **(F)** GSEA of 4 cell death pathway (apoptosis, ferroptosis, necroptosis, and pyroptosis). **(G)** Macrophage infiltration in C1 and C2. **(H, I)** The expression of inhibitory and stimulatory immune ligands in C1 and C2. *p <0.05, **p <0.01, ***p <0.001 and ****p <0.001, ns, no significance.

After analyzing the cophenetic coefficient plot, we determined that K = 2 represented the optimal cluster number (Figures 2A–C). The glioma patients were divided into two efferocytosis patterns, termed Cluster 1 (C1) and Cluster 2 (C2). C1 and C2 gliomas were well separated in the three-dimensional PCA plot, indicating the feasibility of the clustering strategies (Figure 2D). The KM survival plot showed that the median survival time was shorter for C1 than for C2, indicating that the C1 subtype is associated with a poorer prognosis (Figure 2E). We further explored the association with efferocytosis in C1 and C2 cluster by performing GSEA for cell death pathways, analyzing macrophage infiltration, and measuring the expression levels of immune-related ligands. GSEA revealed that upregulation of 4 cell death-related pathways (apoptosis, pyroptosis, ferroptosis, and necroptosis) in the C1 cluster, suggesting that various forms of cell death may be occurring within gliomas of this cluster (Figure 2F). Macrophage immune infiltration analysis revealed that the C1 cluster exhibits a higher level of macrophage infiltration compared to the C2 subtype, encompassing all macrophage states including M0, M1, and M2 (Figure 2G). However, contradictory findings on monocyte infiltration were observed across various computational methods. The results from xCell and CIBERSORT-ABS suggest increased infiltration in the C1 cluster; however, CIBERSORT-RELATIVE detected no significant differences between the clusters. On the other hand, QUANTISEQ identified a higher degree of monocyte infiltration in the C2 cluster. Figures 2H,I shows an elevation in the expression levels of both stimulatory and inhibitory immune ligands in C1. Our analysis indicates that C1 is characterized by the activation of cell death pathways, marked by a significant increase in macrophage infiltration, a rise in the expression of immunosuppressive ligands and a worse prognosis. Based on these findings, we consider C1 as the glioma subtype associated with the process of efferocytosis.

### Partitioning around medoid (PAM) method as the optimal method for predicting patient subtypes

**FIGURE 3:**
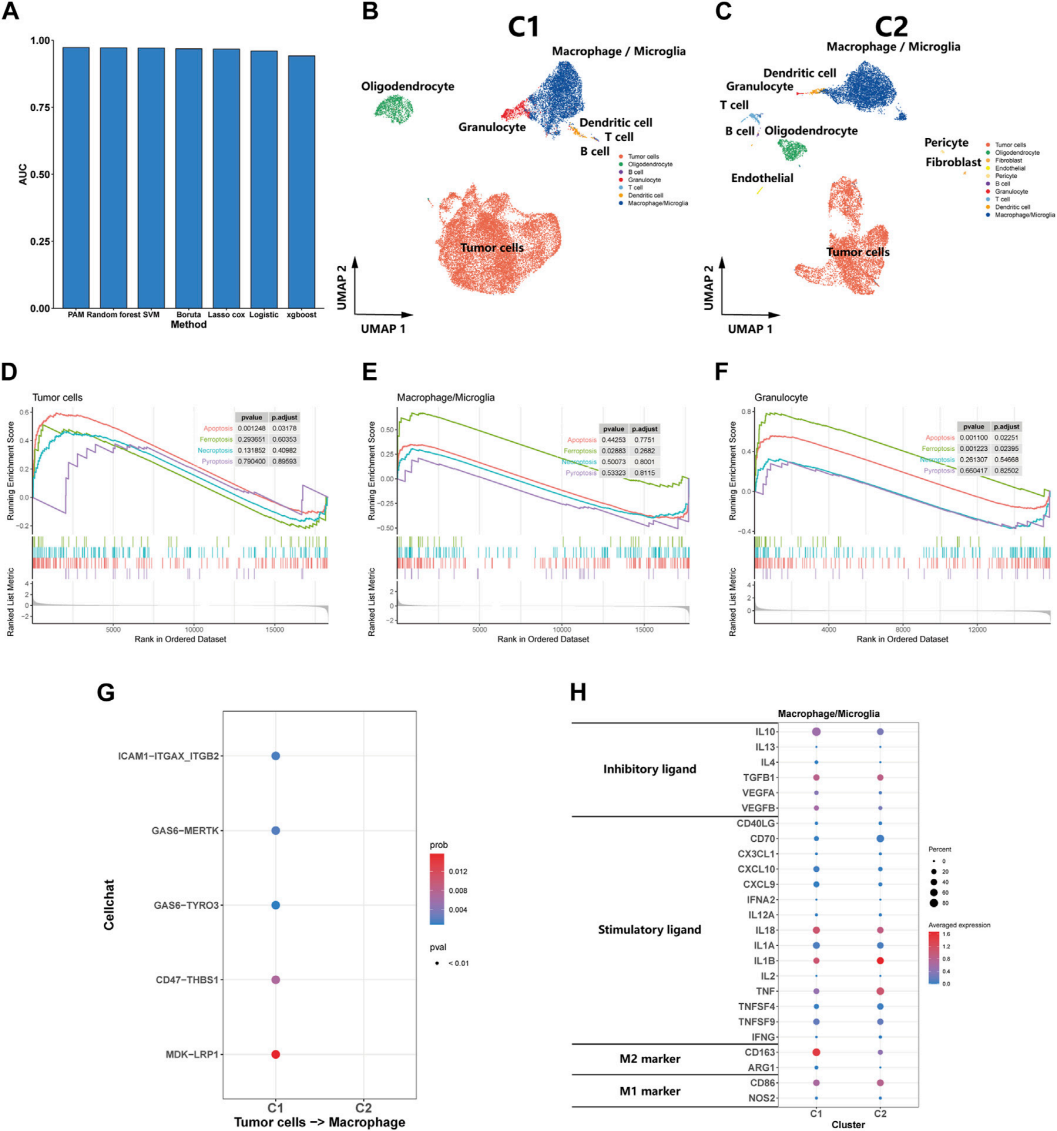
Single-Cell transcriptomic analysis of C1 and C2. **(A)** A bar plot comparing the AUC values of seven distinct predictive methods applied to a test set for patient classifying. **(B, C)** UMAP plot showing the distribution of cells inC1 and C2. **(D, E, F)** GSEA of cell death pathway (apoptosis, ferroptosis, necroptosis, and pyroptosis) for tumor cells, macrophages/microglia and granulocyte. **(G)** Efferocytosis-related intercellular communication in C1 and C2. **(H)** The expression of inhibitory and stimulatory immune ligands, and marker of M1 and M2 in macrophages/microglia of C1 and C2.

We trained models using a dataset constructed from the expression matrix of efferocytosis-related genes, subsequently using these models to predict subtypes in a test set. After evaluating the AUC values of various methods, we discovered the PAM method’s exceptional performance in predicting glioma subtypes, achieving an AUC of 0.973, significantly outperforming other methods (Figure 3A). Consequently, we employed the PAM method for subtype classification in other datasets, ensuring the accuracy and reliability of our predictions.

### Single-cell transcriptomic analysis reveals a stronger association between C1 and efferocytosis

We utilized the PAM method to predict subtypes for eight samples in the Synapse dataset, classifying five as C1 subtype and the remaining three as C2 subtype. Subsequent dimension reduction and analysis of single-cell transcriptomes were performed. The UMAP plot clearly delineated the diverse cellular landscapes of C1 and C2 subtypes (Figures 3B,C). Single-cell GSEA enrichment analysis revealed an upregulation in apoptotic pathways in tumor and granulocyte, as well as ferroptosis pathways in macrophages and neutrophils within the C1 subtype, suggesting various types of cell death occurring (Figures 3D–F). However, we did not observe a significant upregulation in pyroptosis and necroptosis pathways was not observed. CellChat analysis indicated that efferocytosis-related protein interactions were predominantly found in the C1 (Figure 3G). In comparing the macrophages/microglia between C1 and C2 subtypes, we observed a significant increase in the expression of inhibitory immune ligands in the macrophages/microglia of the C1 subtype, along with a rising trend in the markers of M2-type macrophages. These findings suggest that the macrophages/microglia in the C1 subtype tend to exhibit characteristics of M2-type macrophages (Figure 3H). From a single-cell perspective, this finding further emphasizes the C1 subtype’s strong association with the efferocytosis process.

### Functional differences between C1 and C2

**FIGURE 4:**
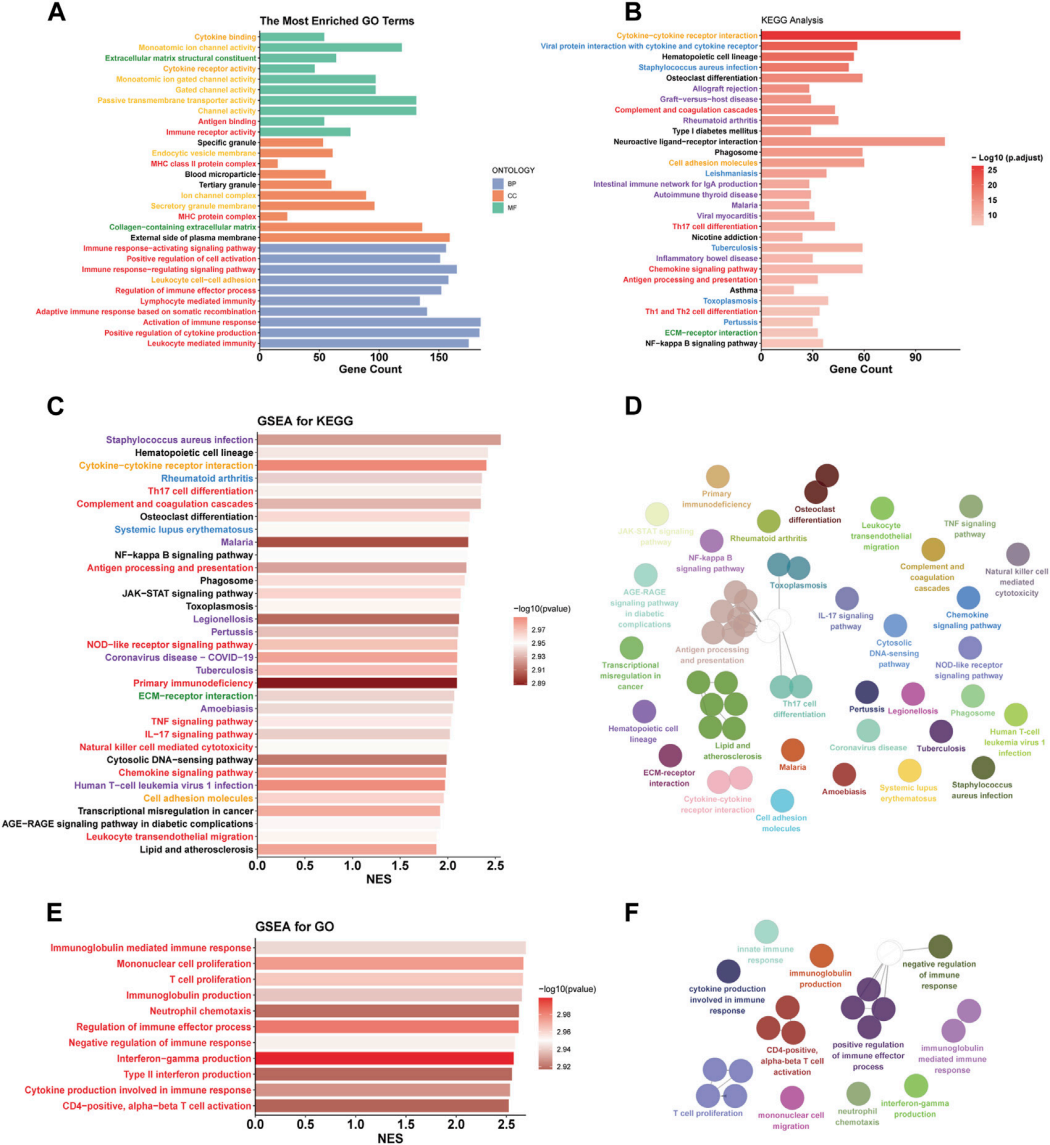
Functional differences between C1 and C2. **(A, B)** GO and KEGG enrichment analysis based on DEGs. **(C, D, E, F)** The simplified top 50 pathways ranked by Normalized Enrichment Score (NES) of the KEGG gene set. **(E, F)** the simplified top 50 pathways ranked by NES of the GO gene set. Category: orange - intercellular communication; yellow - transmembrane transport; red - immune biological process; green - matrix; blue - inflammatory diseases; purple - infectious diseases.

According to the ‘Deseq2′ analysis, C1 exhibited upregulation of 2,796 genes and downregulation of 1,295 genes compared to C2. GO enrichment analysis showed that the DEGs were most enriched in the gene sets associated with intercellular communication (orange labels), transmembrane transport (yellow labels), immune biological process (red labels), and matrix (green labels) (Figure 4A). Immune-related pathways were largely associated with adaptive immunity and T cell activity. Similarly, The results of KEGG enrichment analysis revealed the enrichment of DEGs in pathways related to immunity (red labels), intercellular communication (orange labels), and matrix (green labels) (Figure 4B). Additionally, we found that DEGs were also enriched in pathways related to inflammatory diseases (blue labels) and infectious diseases (purple labels), suggesting that similar biological processes associated with inflammatory and infectious diseases are also occurring in the C1 subtype. To explore the upregulated biological pathways in the C1, we conducted a GSEA. In the KEGG pathway analysis, the C1 subtype predominantly showed upregulation in immunobiological processes, intercellular communication, innate immunity, inflammatory diseases, and infectious diseases, similar to the pathways earlier (Figures 4C,D). In our GO pathway analysis, we observed upregulation in immune activation-related pathways, including adaptive and innate immunity, as well as pathways involving CD4^+^ T cell activation, T cell proliferation, and chemotaxis of monocytes and neutrophils (Figures 4E,F). Notably, there was an upregulation in pathways that negatively regulate immune responses in the C1. This means that both activation and downregulation of the immune response are occurring simultaneously in the C1 subtype.

### Comparing the immune landscapes of C1 and C2 subtypes

**FIGURE 5:**
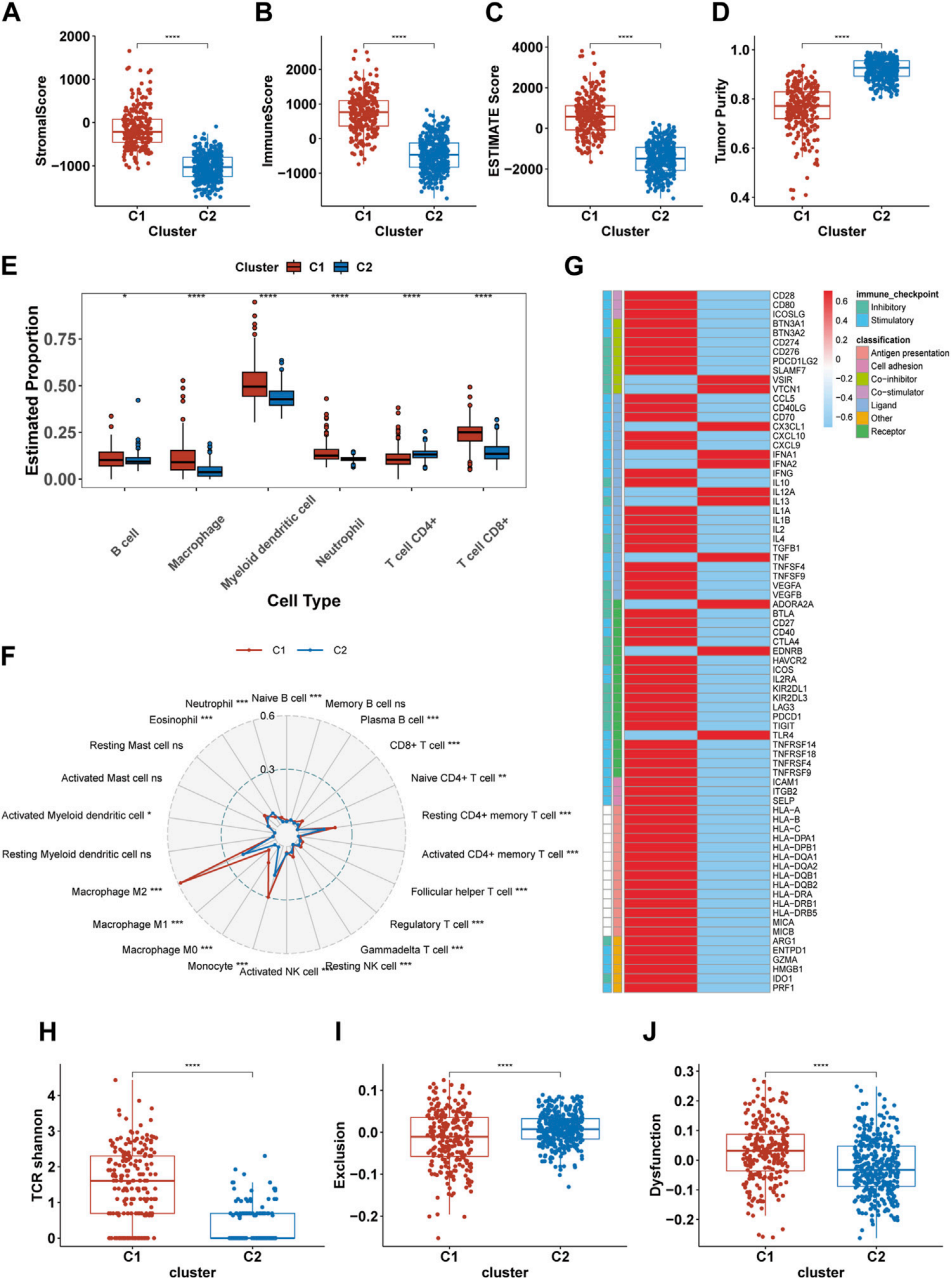
Differences in the immune landscape between C1 and C2. **(A–D)** Comparison of the stromal score, immune score, Estimate score, and tumor purity between C1 and C2. **(E, F)** The infiltration of immune cells in C1 and C2 calculated by TIMER and CIBERSORT-ABS. **(G)** The expression of 75 immunomodulatory genes in C1 and C2. **(H–J)** Comparsion of the TCR shannon **(H)**, Exclusion score **(I)** and Dysfunction score **(J)** between C1 and C2. *p <0.05, **p <0.01, ***p <0.001 and ****p <0.001. ns, no significance.

Previous results suggest significant immunobiological distinctions between the C1 and C2. To further explore these differences in the immune landscape, we first applied the ESTIMATE algorithm. The results indicated higher immune scores, stromal scores, and overall Estimate scores in the C1, coupled with a lower tumor purity, suggesting a richer composition of immune and stromal cells in C1 (Figures 5A–D). Subsequent assessments using TIMER and CIBERSORT-ABS revealed variations in immune cell composition. The TIMER analysis revealed elevated infiltration of macrophages, CD8^+^ T cells, B cells, dendritic cells, and neutrophils in C1, with the exception of CD4^+^ T cells (Figure 5E). CIBERSORT indicated an elevation in antigen-presenting cells (APCs), immunosuppressive cells (like Regulatory T cells (Tregs) and M2 macrophages), and immune-promoting cells (such as CD8^+^ T cells and M1 macrophages), but showed a reduction in NK cell infiltration (Figure 5F). Both algorithms largely aligned, except for CD4^+^ T cells, where TIMER indicated less infiltration in C1 compared to CIBERSORT. Analysis of immune molecular expression demonstrated that most immune molecules in C1, both stimulatory and inhibitory, were expressed at higher levels than in C2 (Figure 5G). Despite the upregulation of immune activation pathways, increased infiltration of pro-inflammatory immune cells, and elevated levels of some stimulatory immune molecules in C1, the prognosis for patients in this cluster remains relatively poor. To investigate this, we compared TCR shannon (reflecting TCR diversity), Exclusion scores, and Dysfunction scores between C1 and C2 (Figures 5H–J). The findings revealed higher TCR shannon and Dysfunction scores in C1, but lower Exclusion scores, suggesting that T cells in C1 gliomas, while diverse and capable of recognizing various tumor antigens, have an impaired ability to eradicate tumor cells.

### Intercellular communication analysis across subtypes

**FIGURE 6:**
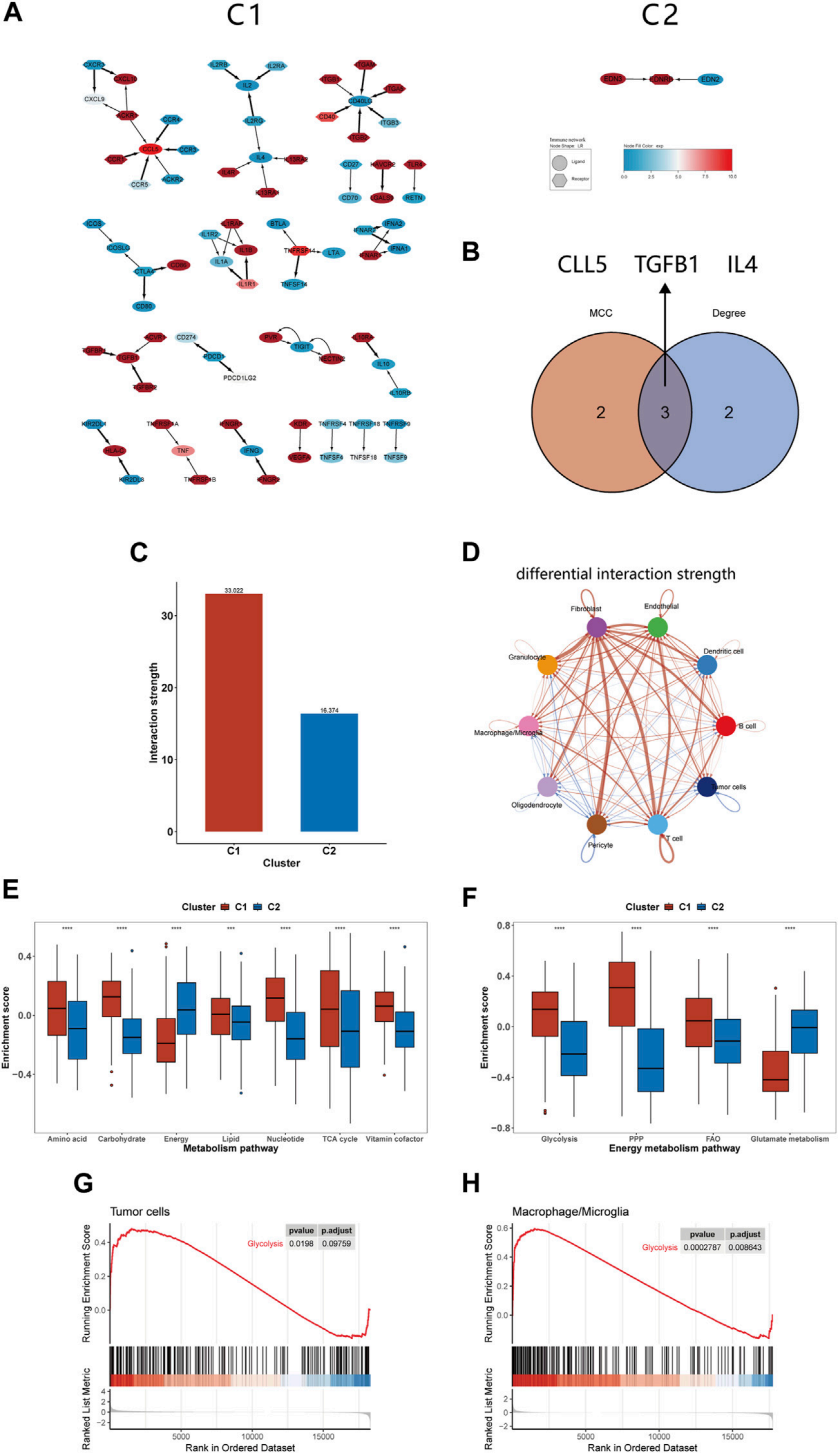
Differences in intercellular communication and substance metabolism between C1 and C2 subtypes. **(A)** Immune network of C1 and C2. **(B)** The 3 hub genes identified by MCC and Degree method. **(C)** The interactions strength of C1 and C2 at the single-cell level. **(D)** Differences in communication strength between different cell types in C1 and C2, with red indicating increased interaction and blue indicating decreased interaction. **(E, F)** Comparison of GSVA enrichment scores for seven metabolism-related pathways and four energy metabolism pathways between C1 and C2. **(G, H)** GSEA of glycolysis pathway for tumor cells and macrophages/microglia. *p <0.05, **p <0.01, ***p <0.001 and ****p <0.001. ns, no significance.

Pathway enrichment analysis strongly indicated differences in intercellular communication between C1 and C2, prompting us to compare their intercellular communication. In analyzing the immune molecular networks of these subtypes, we noted that C1 presented a larger network, suggesting more frequent interactions among its immune molecules and enhanced cellular communication (Figure 6A). By applying MCC and Degree algorithms to analyze hub genes and their intersections, we identified CCL5, TGFB1, and IL4 as key regulators within the C1’s immune network (Figure 6B). Single-cell level assessments confirmed the greater strength of intercellular interactions in C1 compared to C2 (Figure 6C). Figure 6D further elucidates the enhanced interactions between various cell types, notably between tumor cells and macrophages, showing increased activity from tumor cells and reduced influence of macrophages in C1. In comparing the cell communication pathways between subtypes, we observed marked differences: C1 predominantly participates in 27 signaling pathways, while C2 is involved in 35 different pathways (Supplementary Figure S1).

### Differences in metabolic pathways between C1 and C2 subtypes

In our previous analysis, we found differences in substance transport pathways between C1 and C2 subtypes and speculated that macrophage phagocytosis of apoptotic cells alters the tumor metabolism, thereby affecting patient prognosis. Furthermore, the literature about efferocytosis repeatedly emphasizes the link between efferocytosis and metabolic reprogramming, impacting the tumor microenvironment (Boada-Romero et al., 2020; Doran and Yurdagul, 2020; Tajbakhsh et al., 2021). For instance, it is noted that phagocytic cells, after engulfing dead cells, can enhance glycolysis and release lactate; they can also facilitate the conversion of cholesterol to oxysterols, promoting the resolution of inflammation (Morioka et al., 2018; Viaud et al., 2018). Therefore, we continued to study the changes in metabolic pathways between the C1 and C2 subtypes, providing a basis for the application of targeted metabolic drugs to the C1 subtype. Our findings indicated that C1 had higher enrichment scores in most substance metabolism pathways but had lower scores in the energy integration metabolism pathway compared to C2 (Figure 6E). Considering energy metabolism’s crucial role in cancer development, four additional energy-related pathways from Molecular Signatures Database (MsigDB) were analyzed to assess the bioenergetic profiles of the glioma subtypes (Bi et al., 2020; Yu et al., 2021). We observed an upregulation in glycolysis, the pentose phosphate pathway, and fatty acid oxidation pathways in C1, while glutamine metabolism was comparatively downregulated (Figure 6F). Studies shown that reversing glycolysis can induce apoptosis and sensitivity to reactive oxygen species, which is a promising direction for treating gliomas (Caniglia et al., 2021). Moreover, it has been shown in previous research that inhibiting glycolysis in macrophages can suppress efferocytosis, suggesting that targeting glycolysis may represent a novel therapeutic approach (Morioka et al., 2018). We utilized GSEA at the single-cell level to investigate glycolytic pathway changes in tumor cells and macrophages/microglia. The results indicated an upregulation of glycolysis in both of them, which may offer fresh insights into efferocytosis-targeted therapy (Figures 6G,H).

### Genome-wide characteristics of different glioma subtypes

**FIGURE 7:**
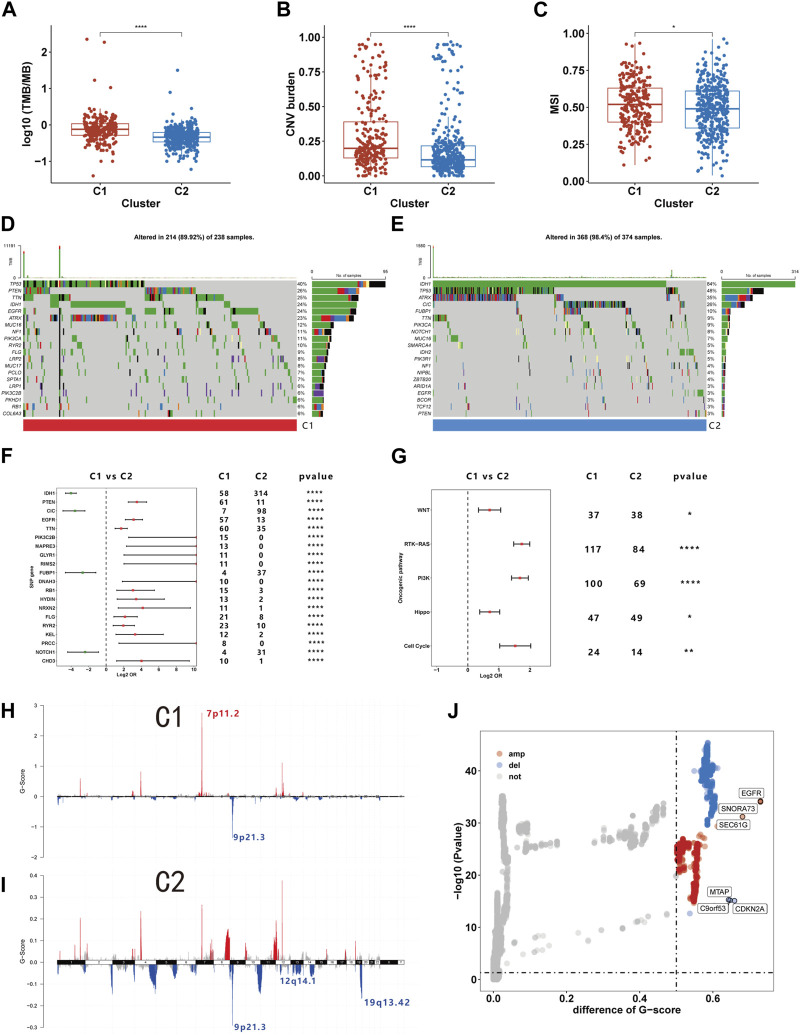
The genome characteristics of C1 and C2. **(A–C)** The TMB **(A)**, CNV burden **(B)**, and MSI **(C)** levels of C1 and C2. **(D, E)** Oncoplot showing the top 20 mutations with the highest frequency in C1 and C2. **(F)** Differential analysis of mutation frequency between C1 and C2. **(G)** Comparison of gene mutations in oncogenic pathways. **(H–I)** The distribution of CNV in C1 and C2. **(J)** Differences in CNV between C1 and C2. *p <0.05, **p <0.01, ***p <0.001 and ****p <0.001. ns, no significance.

Prior research indicates that immune cells can induce genomic instability in the tumor genome through immunoediting (O'Donnell et al., 2019). Given potential immune microenvironment differences between C1 and C2, we investigated genomic variations between these clusters. We observed that the TMB, CNV burden and MSI were all markedly higher in C1 than C2, indicating greater genomic instability in the C1 (Figures 7A–C). Further comparison of somatic mutation genes between C1 and C2 revealed a higher mutation frequency of PTEN, EGFR, and TTN in C1, while IDH, CIC, and FUBP1 mutations were more frequent in C2 (Figure 7F). The oncoplot illustrates the top 20 genes with the highest mutational frequency in C1 and C2 (Figures 7D,E). Additionally, our findings indicate that within ten pathways associated with tumorigenesis, mutations in the RTK-RAS, PI3K, Hippo, Cell cycle, and WNT pathways are more prevalent in C1 (Figure 7G). We also examined CNVs and found that amplifications in chromosome segment 7p11.2 and deletions in 9p21.3 occurred with the highest frequency in C1. C2 exhibited the highest frequency of deletions in chromosome segments 9p21.3, 12q14.1, and 19q13.42 (Figures 7H,I). Subsequent differential analysis revealed that 423 genes were significantly amplified, and 281 genes were deleted in C1 compared to C2 (Figure 7J). Among these CNVs, EGFR and CDNK2A were the most significantly amplified and deleted, respectively. Our analysis revealed distinct genomic characteristics between C1 and C2 subtypes, suggesting a potential link to efferocytosis.

### Correlation between the clusters and sensitivity to antineoplastic drugs

**FIGURE 8:**
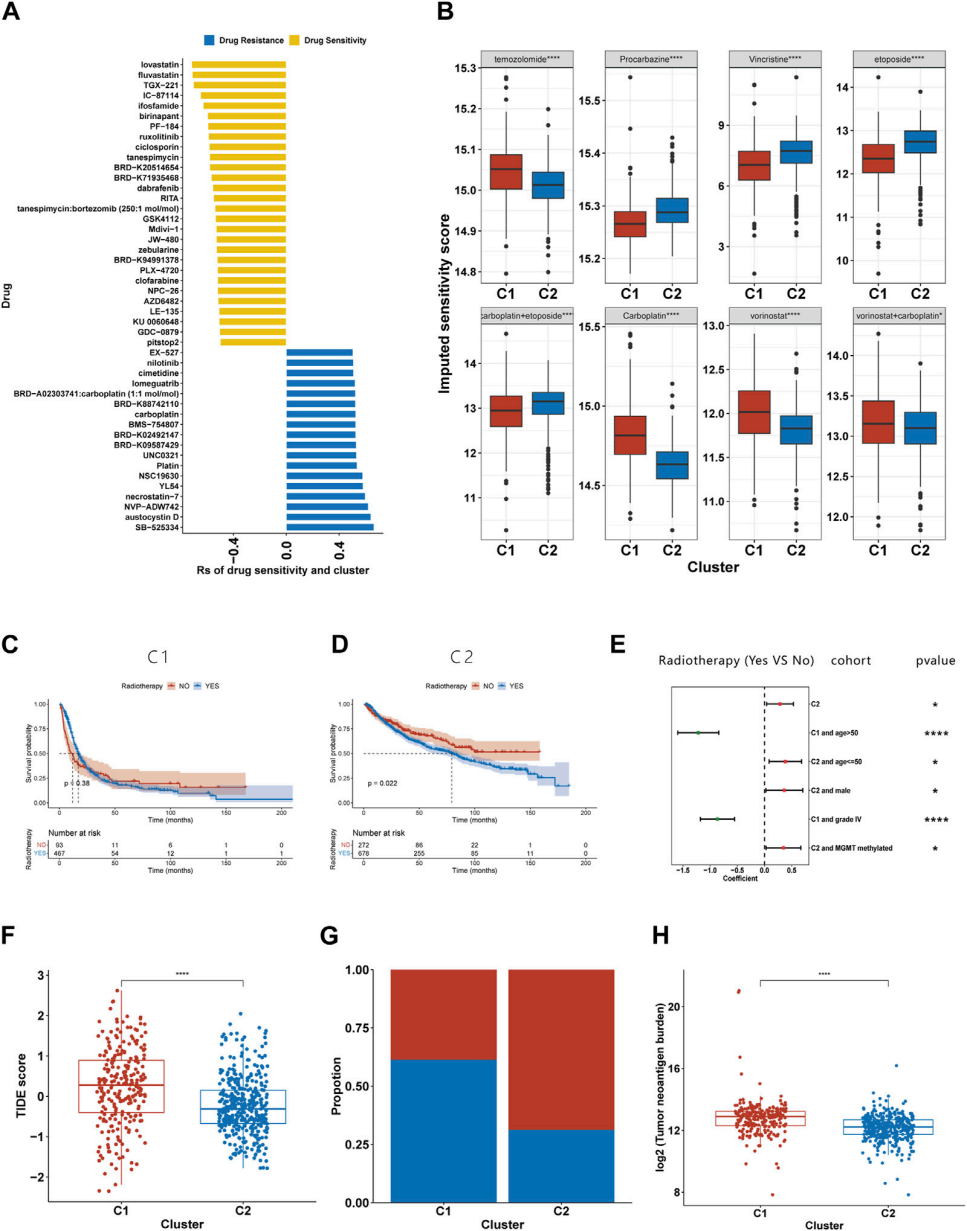
Association between the efferocytosis-related classification and the efficacy of chemoradiotherapy and ICB therapy. **(A)** Correlation between the efferocytosis-related classification and the imputed sensitivity score of 46 drugs. **(B)** The imputed sensitivity score of drugs included in the guidelines for glioma treatment in C1 and C2. **(C–D)** The KM curves for C1 **(C)** and C2 **(D)** who received and did not receive radiotherapy. **(E)** Forest plot showing the risk coefficients for radiotherapy in patients of C1, C1+age≤50, C1+males, C1+MGMT promoter methylationpatients, C2+age>50 and C2+grade IV gliomas group. **(F)** The TIDE scores of patients with C1 and C2. **(F)** The proportion of responders among patients with C1 and C2. **(G)** Compassion of TNB levels between C1 and C2. *p <0.05, **p <0.01, ***p <0.001 and ****p <0.001. ns, no significance.

The imputed sensitivity score of 545 antineoplastic drugs against 623 samples in the TCGA cohorts was calculated using the ‘OncoPredict’ package. Correlation analysis showed that 46 drugs were associated with glioma subtypes (Figure 8A). C1 was relatively resistant to 18 of these drugs and relatively sensitive to the other 28. The 18 drugs to which C1 was relatively resistant targeted a wide range of non-specific molecular entities. Additionally, C1 was relatively sensitive to drugs that targeted BRAF mutation (PLX-4720, dabrafenib and GDC-0879), the PI3K pathway (TGX-221, IC-87114 and AZD6482), HMG-CoA reductase (lovastatin and fluvastatin), and HSP90 (tanespimycin and tanespimycin). By calculating the drug sensitivity score of the eight regimens included in guideline for gliomas, we found that C1 was more sensitive to procarbazine, vincristine, etoposide, and the combination of carboplatin and etoposide, but resistant to temozolomide, carboplatin, vorinostat, and the combination of vorinostat and carboplatin (Figure 8B).

### The relationship between radiotherapy and patient prognosis in different subtypes

Due to the limited number of certain subtypes in specific groups, which prevented meaningful survival analysis, we combined data sets from TCGA, CGGA 693, and CGGA 325 for our analysis. KM survival analysis suggests that the C2 subgroup’s exposure to radiotherapy is associated with a shortened median survival (Figure 8D), while no such relationship was found for the C1 subgroup (Figure 8C). To further explore the link between radiotherapy and survival times in the C1 and C2 subgroups, we stratified glioma patients by clinicopathological characteristics. Our findings reveal that in the C1, older patients or those with grade IV gliomas experienced a prolongation in median survival following radiotherapy (Figure 8E). Conversely, C2 patients under 50 years old, males, or those with MGMT promoter methylation were found to have a shortened median survival post-radiotherapy (Figure 8E).

### Relationship between efferocytosis-related subtype and the response to ICB

Using the TIDE website, we calculated the TIDE score for each sample. Compared to C2, C1 had a higher TIDE score, suggesting it had a poorer response to ICB (Figure 8F). Those with a TIDE score below 0 were categorized as ICB responders, and the proportion of responders was lower in the C1 than in the C2 (Figure 8G). As TMB is used as a biomarker for ICB therapy in some cancers, gliomas with a high TMB have been shown to have a poor response to ICB therapy (Samstein et al., 2019). Our findings of higher TMB levels in C1 support this prediction. Furthermore, our research has shown that C1 exhibits a higher tumor neoantigen burden (TNB), suggesting that a lower quantity of tumor neoantigens may not be the primary reason for the poorer immune response in the C1 (Figure 8H).

### Prognostic prediction accuracy of efferocytosis-related subtype classification

**FIGURE 9:**
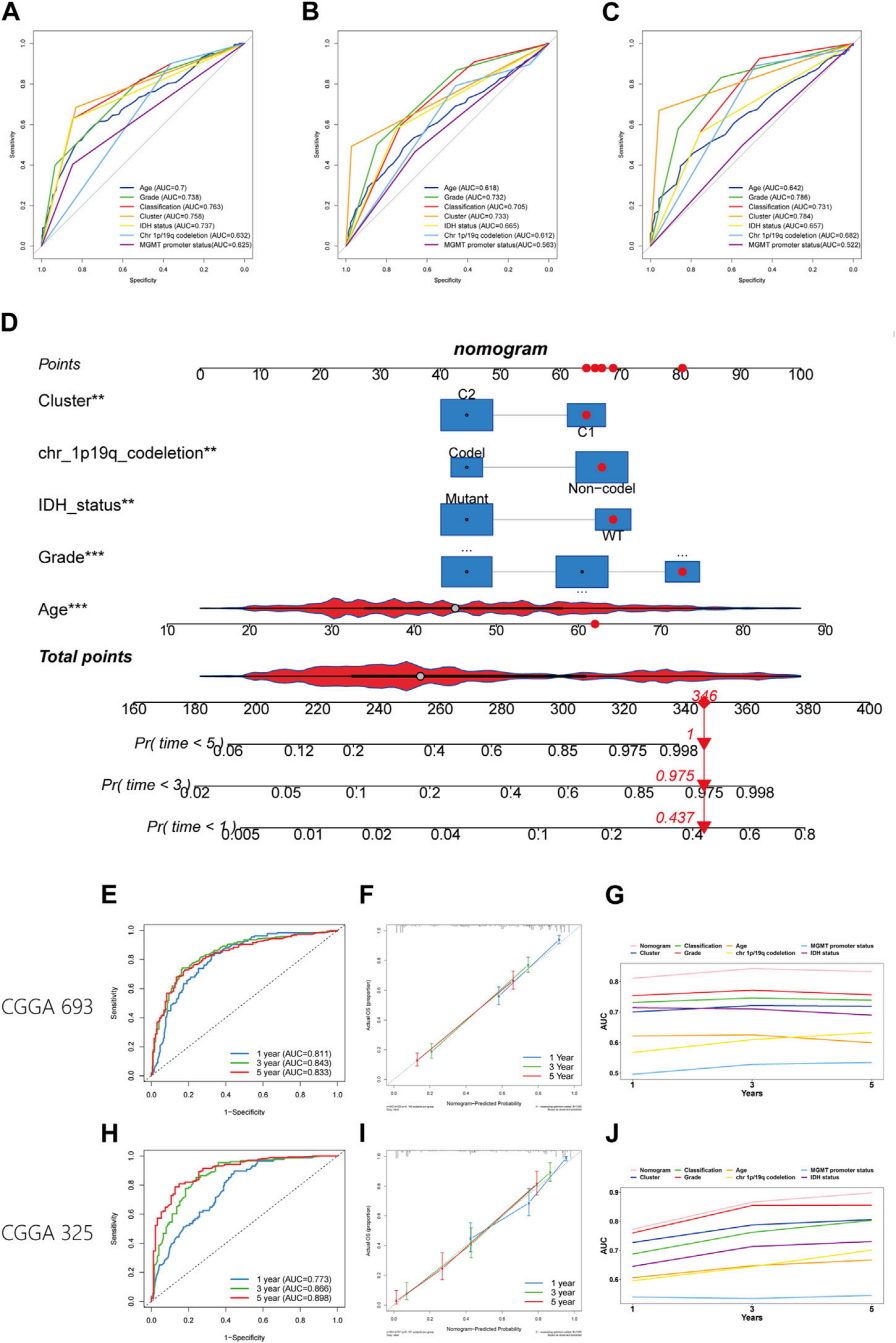
Establishment and verification of a nomogram. **(A–C)** The ROC plot showing the predictive accuracy of efferocytosis-related classification and clinicopathological characteristics in the TCGA, CGGA 693, and CGGA 325 datasets. **(D)** Establishment of the nomogram model. **(E, H)** The ROC plot showing the predictive accuracy of efferocytosis-related classification the CGGA 693, and CGGA 325 datasets within 1-, 3-, and 5-year. **(F, I)** Calibration curve showing the concordance of predicted 1-, 3-, and 5-year survival probability and actual probability for the CGGA 693, and CGGA 325 datasets. **(G, J)** Line chart showing the AUC value of the nomogram, efferocytosis-related classification and clinicopathological characteristics in the TCGA, CGGA 693, and CGGA 325 datasets.

The worse prognosis of C1 compared to C2 suggests that efferocytosis-related subtype classification may have potential for prognostic prediction. We applied the PAM method to classify samples from five datasets (CGGA 325, CGGA 693, Rembrandt, Gravendeel, and Kamoun cohorts) and subsequently conducted survival analyses. The results consistently showed a poorer prognosis for C1 across all datasets (Supplementary Figure S2). In the TCGA, CGGA 693, and CGGA 325 cohorts, where clinical data are more comprehensive, the efferocytosis-related subtype classifications demonstrated good predictive accuracy, with AUC values of 0.758, 0.733, and 0.784, respectively (Figures 9A–C). The AUC values for other clinicopathological features in prognostic prediction are also illustrated in the figures for comparison. Although this classification is not the most predictive among all clinicopathological features, it still offers significant supplementary insight for assessing patient prognosis.

### Establishment and validation of a nomogram model

To better predict patient outcomes, we constructed a prognostic model by integrating efferocytosis-related classifications with clinical-pathological characteristics. Initially, we performed a univariate Cox regression analysis on all variables. The results of univariate Cox regression revealed that prognostic factors for gliomas include cluster, age, grade, classification, MGMT promoter status, chromosome 1p/19q codeletion status, and IDH mutation status (Table 1). Then a multivariate Cox regression analysis identified cluster, age, grade, IDH mutation status, and chromosome 1p/19q codeletion as distinct and independent prognostic factors for gliomas. (Table 1). Afterwards, we train a nomogram model utilizing the factors considered as independent prognostic factors (Figure 9D). This nomogram model was then applied to predict the prognosis of samples in the CGGA 693 and CGGA 325 datasets, calculating the AUC values at 1, 3, and 5-year intervals. The AUC values of the nomogram were 0.811, 0.843, and 0.833 for the CGGA_693 cohort and 0.773, 0.866, and 0.898 for the CGGA_325 cohort, respectively (Figures 9E,H). Furthermore, the calibration curve exhibited a high degree of overlap between predicted and actual survival probabilities (Figures 9F,I). In comparison, the nomogram’s AUC values with those of other characteristics for 1, 3, and 5-year prognostic predictions, as shown in the line charts, indicate that the nomogram model has a significantly stronger prognostic prediction capability than other features (Figures 9G,J). The above results demonstrate that the nomogram exhibits robust prognostic capabilities.

**TABLE 1:** The result of univariate and multivariate Cox analyses in the TCGA dataset.

Variables	Univariate analysis		Multivariate analysis	
	HR (95% CI)	*p*-value	HR (95% CI)	*p*-value
Age (Continuous)	1.070 (1.059–1.081)	<0.001	1.045 (1.032–1.058)	<0.001
Gender (Female vs Male)	1.108 (0.837–1.466)	0.471	-	-
WHO grade				
II	1.000	-	1	-
III	3.313 (2.230–4.922)	<0.001	1.848 (1.212–2.818)	0.00431
IV	21.717 (14.037–33.598)	<0.001	3.245 (1.864–5.648)	<0.001
2021 WHO classification				
O, IDH mutant and 1p/19q codeletion	1.000	-	1	-
A, IDH mutant	1.699 (1.019–2.833)	<0.001	1	-
GBM, IDH wild type	14.228 (8.787–23.040)	<0.001	1	-
IDH mutation (Wild type vs Mutant)	9.936 (7.338–13.451)	<0.001	1.877 (1.143–3.084)	0.0129
Chromosome 1p/19q status (Non-codel vs Codel)	4.249 (2.677–6.746)	<0.001	1.990 (1.171–3.382)	0.0110
MGMTp status (Unmethylated vs Methylated)	3.245 (2.443–4.311)	<0.001	1.259 (0.902–1.757)	0.17634
Cluster (C1 vs C2)	5.583 (4.147–7.516)	<0.001	1.841 (1.244–2.724)	0.00228

## Discussion

Recent studies have elucidated the role of efferocytosis in tumorigenesis and progression, especially in shaping the immune microenvironment (Zhou et al., 2020; Tajbakhsh et al., 2021). For instance, the overexpression of TAM receptors, efferocytosis-related proteins, has been shown to play a pivotal role in macrophage polarization. (Nguyen et al., 2014). Moreover, the use of immunotherapy to block efferocytosis has demonstrated excellent anti-tumor efficacy. Blocking the MerTK receptor in combination with radiotherapy promotes tumor regression and prolongs survival in mice (Wu et al., 2018). Drugs targeting TIM3 are presently under assessment in phase 1 clinical trials (Wu et al., 2018). Due to tumor heterogeneity, immunotherapy may not be suitable for all patients with gliomas. Consequently, numerous studies are currently underway to identify new biomarkers that can keep pace with the rapidly evolving treatment modalities (Arrieta et al., 2023). Our research is also dedicatedly focused on this objective.

Our research aggregated 137 efferocytosi-related genes and analyzed their expression within the TCGA dataset using the NMF algorithm, leading to the identification of two distinct subtypes. Further investigation into enrichment analysis, macrophage infiltration, and immune ligand expression revealed that the C1 subtype is characterized by enhanced cell death pathways, increased macrophage presence, and elevated immunosuppressive ligands, implicating its strong link with efferocytosis. In our analysis, we observed that different algorithms yielded varying proportions for monocyte infiltration. The reason for this may be due to CIBERSORT-RELATIVE and QUANTISEQ calculated the relative infiltration of immune cells, while CIBERSORT-ABS and xCell calculated the absolute quantification of immune cells; therefore, this might account for the contradictory results obtained using different algorithms (Sturm et al., 2019). Additionally, we observed that in the C1 subtype, there is also an increase in the expression of stimulatory immune ligands. We hypothesize two potential reasons for these findings. The occurrence of inflammation could induced by factors like hypoxia or oxidative stress in tumors (Hanahan and Weinberg, 2011). We speculate that in the C1 subtype, this tumor-induced inflammation may initiate cell death and damage, triggering the efferocytosis process and leading to elevated levels of inhibitory immune ligands. The results of our enrichment analysis indicate a concurrent upregulation of immune stimulatory and suppressive pathways in C1, potentially support our hypothesis. Another hypothesis we are considering is that the increased expression of stimulatory ligands may be due to other forms of cell death. Our GSEA enrichment analysis suggests that tumor cells undergo death not only through apoptosis, a relatively gentle process, but also through other forms of cell death, which can provoke a stronger immune response. Necroptosis is known to elicit marked inflammatory responses and adaptive immunity (Lomphithak et al., 2021). And cells release the pro-inflammatory mediators, IL-1β and IL18, when undergoing pyroptosis (Xia et al., 2019). Therefore, the dual upregulation of immune ligands in the C1 could be partially explained by these complex interactions during tumor development.

To investigate the relationship between C1 and efferocytosis at a single-cell level, we classified samples with paired scRNA-seq data into C1 and C2 using the PAM method. We discovered an upregulation in the apoptotic pathways of tumor cells and neutrophils in C1, as well as an increase in the ferroptosis pathways of macrophages/microglia and neutrophils, confirming the presence of multiple forms of cell death in C1. However, our analysis did not reveal any upregulation in pyroptosis and necroptosis pathways across different cell types, a phenomenon that needs further investigation to understand its specific causes. Through CellChat analysis, we discovered that interactions between efferocytosis-related proteins occurred exclusively in the C1 subtype. Additionally, macrophages in C1 exhibited a bias towards the M2 state. Integrating bulk RNA-seq with scRNA-seq analysis, we infer a more robust relationship between C1 and efferocytosis than C2. Five protein-protein interactions within C1 were identified via CellChat analysis. ICAM1 acts as an efferocytosis receptor, while the GAS6 interaction with the TAM receptor facilitates macrophage binding to apoptotic cells (Geng et al., 2017; Wiesolek et al., 2020). THBS1 is linked to efferocytosis in lung injury and IL10 production (Zhao et al., 2014). In atherosclerosis, LRP expression reduces efferocytosis (Doddapattar et al., 2022). These findings may inspire new approaches to glioma immunotherapy by targeting efferocytosis.

To explore the potential mechanisms underlying prognostic differences between the C1 and C2, we undertook a functional enrichment analysis, which revealed differences in intercellular communication, transmembrane transport, immune processes and matrix composition. The pathways of intercellular communication were mainly enriched in cytokine-cytokine receptor interactions and cell adhesion. Based on our previous analysis, C1 is accompanied by an increase in both immunosuppressive and stimulatory ligands, partially explaining the upregulation of cytokine-cytokine receptor interaction pathways. Moreover, the ‘eat me’ signals produced by apoptotic cells, promoting the binding of tumor cells and macrophages, which could partly explain the upregulation of the cell adhesion pathway in C1 (Tajbakhsh et al., 2021). Additionally, the degradation of apoptotic cells by macrophages results in the production of abundant metabolic substances, such as amino acids, lipids, and nucleotides (Han and Ravichandran, 2011). The cellular transport of these metabolites might elucidate the differential activation of transmembrane transport pathways between the two subtypes. Moreover, the transportation of these substances can affect tumor progression and thus potentially impact tumor microenvironment. For example, glycolysis is enhanced in macrophages following the phagocytosis of apoptosis cells, resulting in the production of lactate (Morioka et al., 2018). Studies have shown that the lactate efflux creates a slightly acidic microenvironment and promotes tumor progression via various forms, such as migration, invasion, and angiogenesis (Certo et al., 2021). Thus, efferocytosis might influence patient prognosis by altering the transport of metabolites, thereby changing the tumor microenvironment. The immune-related pathways elevated in C1 predominantly involve the activation and downregulation of immune response, in addition to the stimulation, proliferation, and differentiation of T cells. Recent studies have demonstrated that efferocytosis plays a pivotal role in modulating the differentiation of T cells, potentially impacting immune responses. After macrophages phagocytose apoptotic cells, the released anti-inflammatory mediators can promote Treg differentiation (Blander, 2017). In turn, Tregs can enhance efferocytosis, facilitating anti-inflammatory tissue repair (Proto et al., 2018). Moreover, steroids synthesized by macrophages during efferocytosis can induce differentiation of both Tregs and Th2 cells (Proto et al., 2018). Our analysis of pathway enrichment has further developed our initial hypothesis regarding the simultaneous increase in stimulatory and inhibitory immune molecules in C1. We assume that C1 undergo various forms of cellular damage and death, triggering an immune response. The products and cytokines of apoptotic tumor cells attract monocytes to the tumor. After macrophages phagocytose apoptotic tumor cells, they not only alter the tumor microenvironment’s metabolism through digestion and substance transport but also present antigens to T cells, thereby stimulating T cell activation and proliferation. However, these macrophages also release immunosuppressive ligands after ingesting apoptotic cells, leading to Th2 and Treg differentiation and suppressing the immune response. This may finally result in a complicated immune microenvironment with simultaneous upregulation of inflammatory and anti-inflammatory pathways. Based on the results of bioinformatics analysis, we have made the above assumptions, which still require further experimental validation.

To better understand the two subtypes, we conducted a further assessment of the differences in the tumor microenvironments of C1 and C2. The result of ESTIMATE indicates a higher infiltration of immune and stromal cells and lower tumor purity in C1 compared to C2. The increased infiltration of stromal and immune cells may be due to the “find me” signals from apoptotic cells and the chemokines released by macrophage (Wei et al., 2020). Immune cells infiltration analysis indicated an increase in both immunosuppressive and immune-stimulatory cells in C1. The rise in immunosuppressive cells may account for the poorer prognosis observed in C1 patients. However, the elevated numbers of anti-tumor cells, including M1 macrophages and CD8^+^ T cells, did not improve patient survival. Though C1 exhibited increased infiltration of CD8^+^ T cells, serveral studies indicate their dysfunction, leading to an inadequate production of cytotoxic factors during tumor development and progression. (Wei et al., 2020). Our analysis of T cells supports this view. We observed that C1 has higher TCR Shannon and Dysfunction scores and lower Exclusion scores compared to C2. This suggests that T cells recognize tumor antigens and infiltrate the tumor, but their ability to kill tumor cells is impaired. As for M1 macrophages, a study showed that M1 macrophages promote tumorigenesis by creating a mutagenic microenvironment (Salmaninejad et al., 2019). Moreover, a research suggests that TNF-α, which is released by M1 type macrophages, plays a role in facilitating tumor progression (Gong et al., 2022). This may partly explain the worse prognosis for the C1 subtype, which is associated with a higher infiltration of M1 macrophages. Further immune landscape analysis support our initial hypothesis: the C1 subtype exists within a complex immune microenvironment characterized by simultaneous immune suppression and activation. Additionally, we discovered dysfunction in T cells within C1, which might explain why increased CD8^+^ T cell infiltration in gliomas does not translate into better patient prognosis.

Intercellular communication analysis revealed a larger and more active immune network in C1 compared to C2. Our study identified CCL5, TGFB1, and IL4 as hub genes within the C1 immune network. Macrophages release TGF-β after phagocytosing apoptotic cells, promoting tissue repair, while IL4 is essential for their phagocytic function (Fadok et al., 1998; Bosurgi et al., 2017). The relationship between CCL5 and efferocytosis remains unclear and warrants further investigation. Exploring how these hub genes interact with efferocytosis and pursuing the development of drugs to suppress this mechanism might offer a new avenue for research and therapy. Additionally, our single-cell level CellChat analysis of C1 and C2 also showed enhanced intercellular communication, consistent with our enrichment analysis results.

Our analysis revealed a distinct metabolic alteration in C1 compared to C2, characterized by a downregulation of energy metabolism pathways and an upregulation of other substance metabolism pathways. This finding implies that C1 may have undergone metabolic reprogramming, reducing its energy requirements and enhancing other forms of metabolism to produce materials necessary for cell proliferation (Bi et al., 2020). Moreover, discovered that the C1 predominantly relies on glycolysis, the pentose phosphate pathway, and fatty acid oxidation for energy metabolism, while reducing its dependency on glutamine/glutamate. Additionally, at the single-cell level, we observed an upregulation of the glycolysis pathway in both tumor cells and macrophages/microglia within C1. Furthermore, a study has shown that inhibiting glycolysis in phagocytic cells can suppress efferocytosis (Morioka et al., 2018). Research also indicates that blocking the lactate released by efferocytotic cells induces IL10 expression in bone marrow-derived macrophages, leading to anti-inflammatory effects (Morioka et al., 2018). Therefore, employing glycolysis-reversing drugs treating C1 patient could not only suppress efferocytosis within the tumor but also reduce the tumor cells’ energy supply, making it a promising therapeutic approach (Caniglia et al., 2021).

On a genomic level, our findings indicate that C1 exhibits elevated TMB, CNV burden, and MSI, suggesting a pronounced genomic instability within this subtype. Cells with genomic instability are more prone to either evolution or death (Sulkowski et al., 2020). We speculate that in the C1 subtype, the death of genomically unstable tumor cells might be a contributing factor triggering efferocytosis. And a study has shown that under immunological pressure, tumor cells can undergo immune editing, leading to genomic alterations (O'Donnell et al., 2019). Changes in the immune microenvironment induced by efferocytosis might play a role in affecting the genomic instability within the C1. Moreover, we observed a higher mutation frequency of EGFR, PTEN, and TTN in C1, along with chromosomal amplification at 7p11.2 and deletion at 9p21.3, which involve genes EGFR and CDKN2A respectively. Compared to C2, C1 exhibited higher mutation frequencies in pathways including WNT, RTK-RAS, PI3K, Hippo, and the Cell Cycle. The relationship between these genetic aberrations and efferocytosis remains unreported, highlighting an area ripe for further exploration.

Next, we explored the clinical application of the efferocytosis-related subtype classification. OncoPredict results indicated that C1 patients were more sensitive to procanazine, vincristine, etoposide, and carboplatin, and the carboplatin-etoposide combination. While C2 patients showed greater sensitivity to temozolomide, carboplatin, vorinostat, and the carboplatin-vorinostat combination. Therefore, among the eight regimens recommended in the guideline, Procarbazine + CCNU + Vincristine (PCV) regimens and etoposide may be most suitable for C1 patients, while temozolomide, carboplatin, vorinostat, and the vorinostat-carboplatin combination might be more beneficial for C2 patients. Moreover, the efferocytosis-related subtype classification correlated with the imputed sensitivity score of certain antineoplastic drugs. Compared to C2 patients, antineoplastic drugs targeting BRAF mutations, the PI3K pathways, and HSP90 seemed more effective in C1 patients. This effectiveness may be attributed to the higher frequency of mutations in the PI3K pathway involvement in C1, thereby supporting the accuracy of this predictive method.

Our study also revealed differences in the efficacy of radiotherapy between C1 and C2 patients. We observed that C1 patients did not show a significant difference in survival time with or without radiotherapy. In contrast, C2 patients who underwent radiotherapy had a shorter median survival time compared to those who did not receive it. This may be due to C2 patients’ relative sensitivity to temozolomide and the adverse side effects of radiotherapy. Moreover, research indicates that tissue damage from radiation therapy may promote tumor progression during tissue repair (Nolan et al., 2022). Particularly in the case of C2 tumors, which have a lower level of efferocytosis, the damage from radiotherapy might trigger efferocytosis, releasing tissue repair factors that may further facilitate tumor growth and spread. However, this finding was not observed in patients with different clinicopathological characteristics. C2 patients under 50 years old, males, or those with MGMT promoter methylation, had a shortened median survival post-radiotherapy. Conversely, C1 patients over 50 or those with grade IV gliomas benefited from radiotherapy. Considering the high sensitivity of patients with MGMT promoter methylation to temozolomide, we speculate that in C2, the tumor growth could be control by temozolomide. The addition of radiotherapy might lead to adverse side effects, potentially worsening the prognosis for these C2 patients. However, the underlying mechanism accounting for the differences in radiotherapy efficacy for other subgroups based on different clinical characteristics remains unclear. Further research is needed to explore the results.

Moreover, the efferocytosis-related classification was associated with the response to ICB. C1 patients exhibited a poorer response to ICB. Our findings regarding the immune landscape indicate T-cell dysfunction in the C1. This dysfunction may likely contribute to the poor response of C1 patients to ICB treatment. Consequently, C1 patients might require the combination treatment discussed earlier, integrating radiotherapy with drugs blocking TIM3 and PD-1, to stimulate the tumor’s internal immune response, activate T-cells, and kill tumor cells (Kim et al., 2017). Such a combination therapy holds promise as a potential strategy for C1 patients.

Given the poorer prognosis of C1 patients compared to those in C2, we speculate that this classification could be a robust prognostic indicator. By classifying samples from five datasets using PAM, we consistently observed that the prognosis for C1 was worse than for C2, confirming the classification’s predictive capability across external datasets. When comparing this classification’s prognostic accuracy against clinical features of glioma in the TCGA, CGGA 693, and CGGA 325 datasets, it demonstrated good performance. However, in some datasets, its predictive accuracy was not as high as glioma grade or WHO classification, yet it may still serves as a valuable supplementary prognostic factor. We then developed a more powerful nomogram model based on the independent prognostic factors identified in the TCGA dataset, aiming to enhance the accuracy of patient prognosis prediction. The nomogram demonstrated robust predictive ability in the CGGA 693 and CGGA 325 datasets. We hope that this nomogram will provide a better understanding of the prognosis for glioma patients.

There are several limitations to our study. First, some results were obtained via a previously published algorithm. Although these algorithms applied are widely recognized, further experimental verification is required to support the results. Second, this was a retrospective study based on data available in a public databases. Thus, a prospective study is needed to validate the findings. Finally, our understanding of the relationship between efferocytosis-related subtypes and the immune landscape, intercellular communication, substance metabolism and genomics is currently hypothetical. Future work will require extensive experimentation to explore the regulatory mechanisms of efferocytosis within these subtypes and their impact on the tumor microenvironment. Identifying related targets and developing drugs to improve prognosis for these patients is our next goal.

## Conclusion

In this study, we defined two subtypes of glioma based on the expression of 137 efferocytosis-related genes. We explored the differences between these two subtypes in terms of immune landscape, intercellular communication, substance metabolism, and genomic variations, and hypothesized their connections with efferocytosis. Furthermore, we discovered that the classification of efferocytosis-related subtypes not only serves as a robust prognostic indicator but also correlates with the efficacy of radiotherapy, chemotherapy, and ICB treatments. While our research may not be fully comprehensive, we hope the findings of our study can assist clinical decision-making for patients with glioma and provide novel insights for research, contributing to the development of personalized therapy.

## Data Availability

The datasets presented in this study can be found in online repositories. The names of the repository/repositories and accession number(s) can be found in the article/Supplementary Material.

## Supplementary material

SUPPLEMENTARY FIGURE S1 The pathways with frequent interaction within the C1 and C2 subtypes.

SUPPLEMENTARY FIGURE S2 The KM curve shows the prognostic value of efferocytosis-related classification in the CGGA 693, CGGA 325, Rembrant, Gravendeel, and Kamoun cohorts.

SUPPLEMENTARY TABLE S1 The list of the efferocytosis-related genes.
