# Supplementary figures and images for: Identification of efferocytosis-related subtypes in gliomas and elucidating their characteristics and clinical significance
Source: Front Cell Dev Biol. 2023 Dec 13;11:1295891. doi: 10.3389/fcell.2023.1295891 (PMC10757721; doi:10.3389/fcell.2023.1295891)

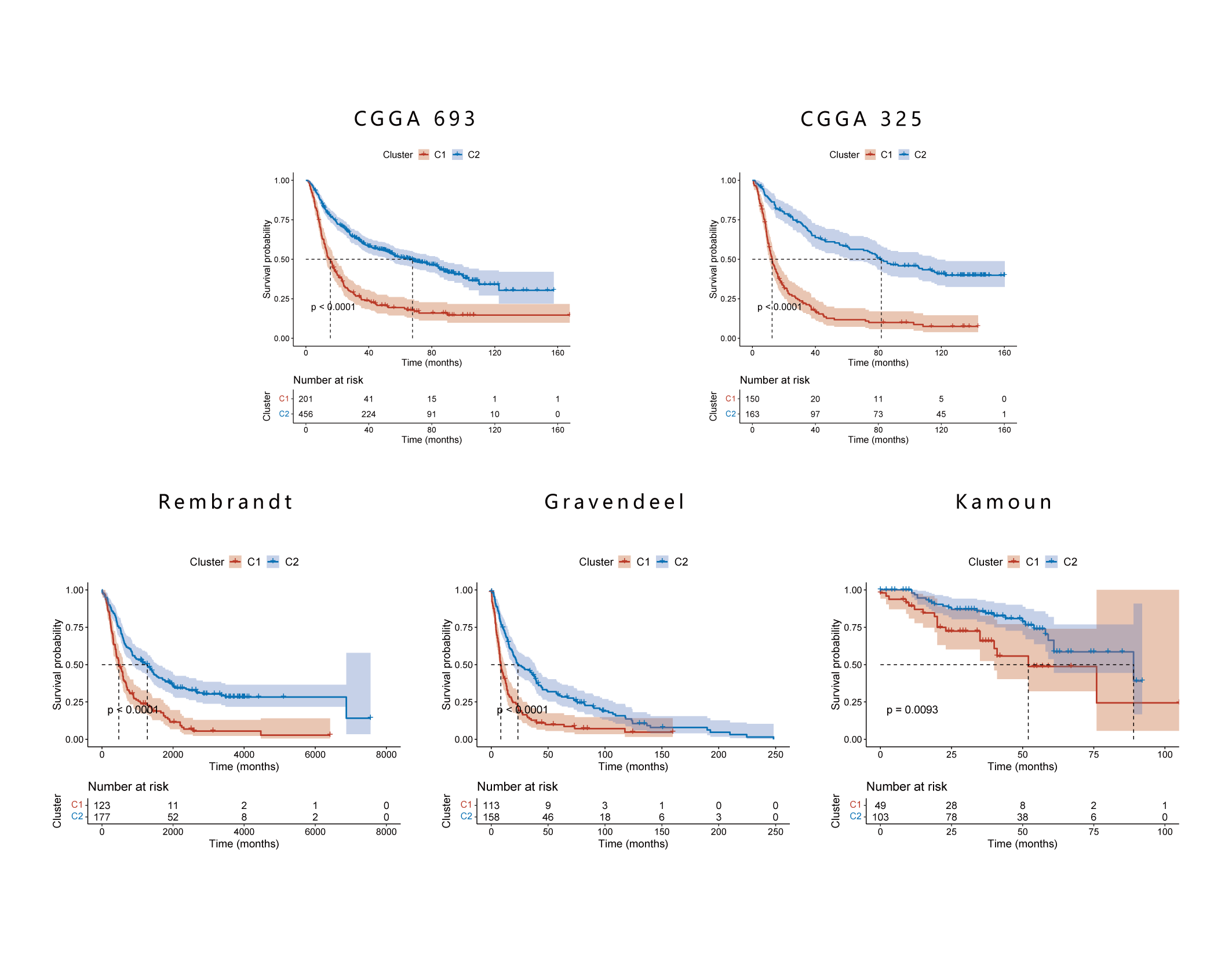

Supplement: Supplementary file 2 [file Image2.TIF]

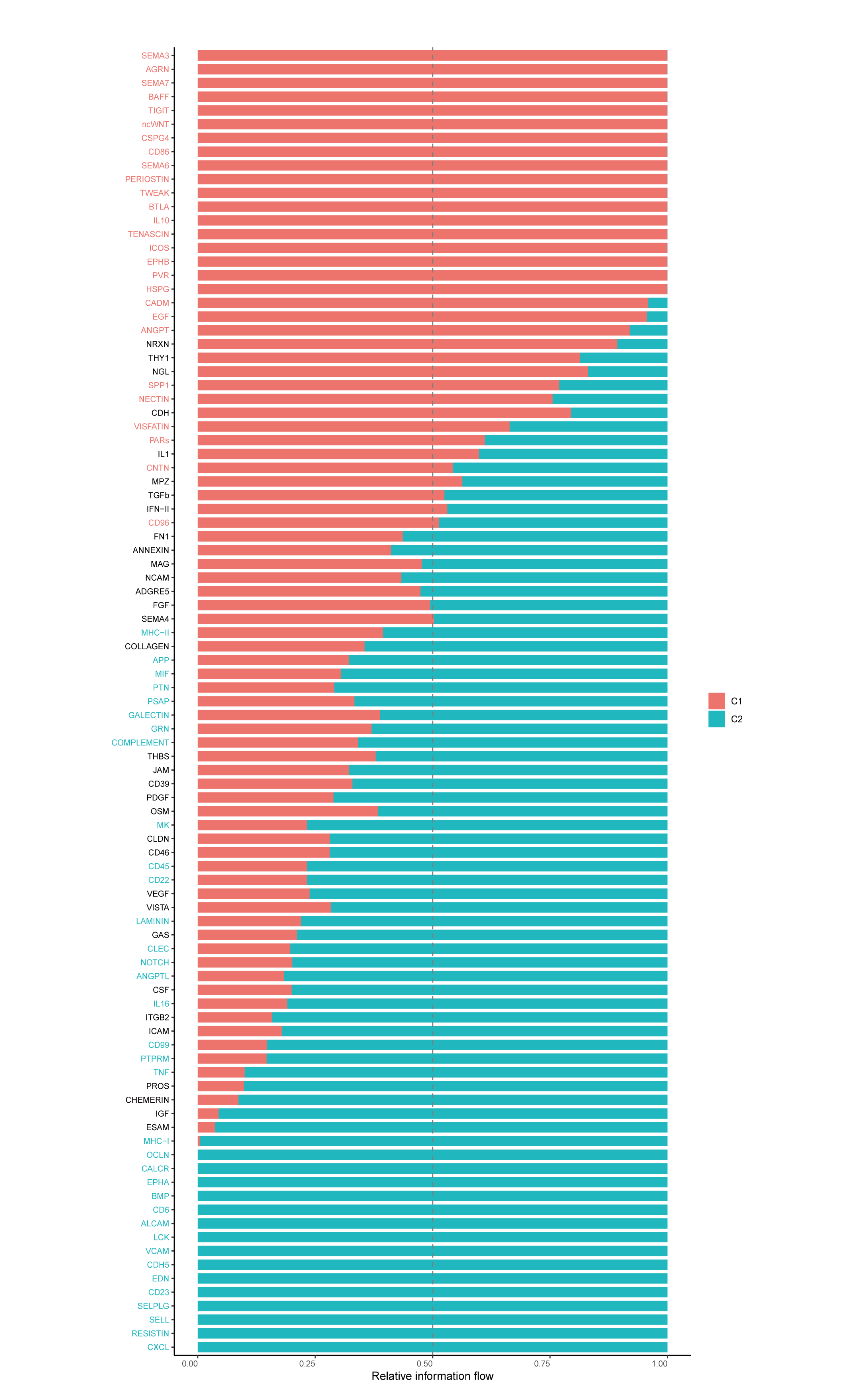

Supplement: Supplementary file 3 [file Image1.TIF]
